# 

**Published:** 1892-05-07

**Authors:** 


					May 7, 1892. THE HOSPITAL.
CONTENTS.
Jhe Hospitals and Hospital Saturday ?
81 I
P&^ingr ropics,-
-Infbri tei
S2
Doctor a Armchair.-
-Anarchism in Physio-
83
Jfasro* versus comfort.-
-A Sco^tis*1 Sketoh
84
?H?>JLcal History irom the Earliest Times.
By E. T.
Wiihington, M.A., M.B.Oxon.
VII.?Ohaldcan and Persian
Me ioine
85
Jk? Story of the Insane from Year to Tear
86
body's Page
86
Annotations
-H rte Civilisation in 8hntland and Wales.?The
Re n n of he Sea Serpent.?Thankless Unpopularity-
87
gotes and Hews-
88
Student of Medicine. ? Students' Bookshelf ? Hospital
News? Appointments?Vacancies.
A round the Hospita's-
-Scraps and Gleaning* ?
89
The Practitioner's Mirror and <*-eci ospeot-
Medicinb -
Toe i'ie* mentof No*turu?l Incontineno1)-
- Ohronio
UL ?pit of *he L?cr?>
90
Practitioner's Bookshel?. -
- Diseeotion Guides -
- Aids to
Ge *ral P-ithVo/y
91
N w Dihtgs Appliances, and Things Mkdical?
-A NoUe'eu
Pail
91
The institutional Workshop ?
Hospital Ai minirtiution :
An Tflpal 8cheme of Oo-oirration
93
Ths Wobsshof BOoKSHEL*.
Two Haud boons on Ho- bo Drain*
94
Fun iron* and Fittim9s.
?A Msdel Sl'p Sink ?
94
Hospital Recohstbdctioh.-
-Ths BadoliSa Infirmary
95
EXTRA SUPPLEMENT THE NURSING MIRROR.
En Passant.?London 8 hoot of Medicine for Women?Corres-
pondents?Woolwich District Nursintr?NurBes for India?
Samaritan Booiety at St. Thomas's?The Oare of the Insane
in New York City?Lowestoft Hospital?The Oare of the
.^J'FeeMe Minded" - - ? - ? * _? "
xxxvii
"VentUationT, Disinfection, and Diet. By P.? -
Bmith, M.D. IV.?Artificial Ventilation ? Extraction _ xxxyiii
SlttSuitetf of the lAsane. By A Medical Oihmb. Why ^
^ Tra niuB is MpoesB *y (continued) ? _ xxxix
Wants and Workers
Por Beading to the Sick.?"8iok and Afflicted" ? ? xxxix
Nursingr Ho >.es IV - K ngr's 0 lln?e Hospital ? xl
Z nana Bible and Me ioal Mission xl
Facts and Foundlings   xli
Registration of Mid drives Bill ...... xli
?moag'ihe In titn    xli
Everybody's Opinio a. Sompthiojj mora ?bout Holidays
fromaNarge?N irt.es' Ho'idwa ? A D.ffioult.Oaii ? xlii
Where to Go -U"ote? a"d Queries  xlii
Four Months in a Hospital Ward (eont<nu?d) ? ? ? xliii
Off Duty: A True Sto y ot Flows s (concluded) ... xlir

				

